# Chitosan Coating Enriched With *Ruta graveolens* L. Essential Oil Reduces Postharvest Anthracnose of Papaya (*Carica papaya* L.) and Modulates Defense-Related Gene Expression

**DOI:** 10.3389/fpls.2021.765806

**Published:** 2021-11-11

**Authors:** Lucia Landi, Yeimmy Peralta-Ruiz, Clemencia Chaves-López, Gianfranco Romanazzi

**Affiliations:** ^1^Department of Agricultural, Food and Environmental Sciences, Marche Polytechnic University, Ancona, Italy; ^2^Faculty of Bioscience and Technology for Food, Agriculture and Environment, University of Teramo, Teramo, Italy; ^3^Facultad de Ingeniería, Programa de Ingeniería Agroindustrial, Universidad del Atlántico, Puerto Colombia, Colombia

**Keywords:** chitosan, essential oils, gene expression, induced resistance, RT-qPCR

## Abstract

Anthracnose of papaya (*Carica papaya* L.) caused by the fungus *Colletotrichum* spp. is one of the most economically important postharvest diseases. Coating with chitosan (CS) and *Ruta graveolens* essential oil (REO) might represent a novel eco-friendly method to prevent postharvest anthracnose infection. These compounds show both antimicrobial and eliciting activities, although the molecular mechanisms in papaya have not been investigated to date. In this study, the effectiveness of CS and REO alone and combined (CS-REO) on postharvest anthracnose of papaya fruit during storage were investigated, along with the expression of selected genes involved in plant defense mechanisms. Anthracnose incidence was reduced with CS, REO, and CS-REO emulsions after 9 days storage at 25°C, by 8, 21, and 37%, respectively, with disease severity reduced by 22, 29, and 44%, respectively. Thus, McKinney’s decay index was reduced by 22, 30, and 44%, respectively. A protocol based on reverse transcription quantitative real-time PCR (RT-qPCR) was validated for 17 papaya target genes linked to signaling pathways that regulate plant defense, pathogenesis-related protein, cell wall-degrading enzymes, oxidative stress, abiotic stress, and the phenylpropanoid pathway. CS induced gene upregulation mainly at 6 h posttreatment (hpt) and 48 hpt, while REO induced the highest upregulation at 0.5 hpt, which then decreased over time. Furthermore, CS-REO treatment delayed gene upregulation by REO alone, from 0.5 to 6 hpt, and kept that longer over time. This study suggests that CS stabilizes the volatile and/or hydrophobic substances of highly reactive essential oils. The additive effects of CS and REO were able to reduce postharvest decay and affect gene expression in papaya fruit.

## Introduction

Papaya (*Carica papaya*) is a fruit cultivated in tropical and subtropical regions but appreciated worldwide. It is known for its high nutritional and economic potential (Parven et al., 2020). Papaya fruit is rich in vitamins A and C, and in gallic acid, alkaloids, flavonoids, other phenolic compounds, and papain, an enzyme with extensive uses in the pharmaceutical, medical, and food industries (Brishti et al., 2013; Jarisarapurin et al., 2019). However, being a climacteric fruit, it is subject to intense metabolic activity, fast maturation, high susceptibility to fungal diseases, and short shelf life (Batista et al., 2020).

The most common fungal disease of papaya fruit is anthracnose, which is caused by *Colletotrichum* spp. and can result in 30–50% postharvest losses (Gunathilake et al., 2018). Several technologies have been used to extend the postharvest shelf life of fruit, including fungicides, low-temperature storage, thermal processing, diverse packaging conditions, and preserving compounds obtained from natural sources that are “generally recognized as safe” (Droby and Wisniewski, 2018). Exports of papaya fruit have been projected to grow at 1.7% per year over the medium term by the United Nations Food and Agriculture Organization, to potentially reach 3,18,000 t of fruit by 2028 (Food and Agricultural Organization of the United Nations, 2020). This, thus, indicates the opportunity for significant trade growth.

Chitosan (CS) is a natural biocompatible polysaccharide that is known to be an effective eco-friendly alternative to synthetic fungicides (Mutjaba et al., 2019; Rajestary et al., 2021). In recent years, CS has been used as a natural fungicide and plant defense booster based on its antimicrobial, film-forming, and eliciting defense activities (Feliziani et al., 2015; Romanazzi et al., 2018; Duan et al., 2019). Because of its film-forming properties, it can be used as a coating for many fruits and vegetables, to create a modified atmosphere around the product that prolongs the shelf life and retains the physicochemical and sensory properties (Valencia-Chamorro et al., 2011; Romanazzi et al., 2018). CS can elicit defense mechanisms of papaya (Ali et al., 2012), peach (Ma et al., 2013), banana (Hernández-Ibáñez et al., 2013), strawberry (Landi et al., 2014, 2017), orange (Coqueiro et al., 2015), avocado (Obianom et al., 2019), and grapes (Zhang Z. et al., 2020).

The physicochemical properties of CS relate to its hydrophilic nature, and these can be reinforced with the introduction of hydrophobic compounds, such as some essential oils (EOs). EOs such as those obtained from *Cymbopogon citratus*, *Origanum vulgare*, and *Thymus capitatus*, among others, have shown encouraging benefits when used as postharvest strategies for food preservation (Pisoschi et al., 2018; Sivakumar and Romanazzi, 2019). However, EOs are highly volatile, and thus their persistence on products when applied can be low (Alonso-Gato et al., 2021). This inconvenience could be reduced by encapsulating such EOs in polymers such as CS to potentially use them as alternatives to traditional fungicides (Rodríguez et al., 2016). In recent years, the use of CS-based composite coatings that incorporate EOs has been proposed as postharvest treatments for fruit (Yuan et al., 2016; Grande-Tovar et al., 2018). In most cases, these studies have confirmed the conservation of fruit physicochemical properties, inhibition of pathogenic microorganisms, and the extension of shelf life of fruit (Munhuweyi et al., 2017; Lima Oliveira et al., 2018). More recently, it was reported that an emulsion formed from 2% CS combined with different concentrations of *Ruta graveolens* (rue) EO (REO) has efficacy as a coating of postharvest fruit, with extended shelf life seen for guava (Grande Tovar et al., 2019), gooseberry (González-Locarno et al., 2020), tomato (Peralta-Ruiz et al., 2020b), pear (Peralta-Ruiz et al., 2021), and papaya (Peralta-Ruiz et al., 2020a). However, to our knowledge, the mechanisms associated with the protective effect induced by REO and CS-REO treatments in fruit are not well understood. It is well known that plant immune regulation is a defensive strategy of plants for protection against pathogen invasion, in addition, some substances can induce plant autoimmunity regulation mechanisms. In this context as mentioned above, CS is well known as an elicitor of plant defense responses, while the defense mechanism induced by REO and CS-REO combination was not investigated. Thus, the objectives of this study were to determine if the treatments of the papaya fruit with 0.5% CS, 0.5% REO, and 0.5% CS-REO combination were able to induce resistance and/or the activation of plant defense mechanisms, testing the early gene expression of key genes involved in plant response against biotic and abiotic stress. Furthermore, the effectiveness of postharvest control of anthracnose following treatments was analyzed.

## Materials and Methods

### Fruit Samples

Papaya fruits cultivated in Brazil were obtained from a local market in Ancona (Marche region, Italy) at the third maturation stage according to the maturity scale proposed by Santamaría et al. (2009). Fruits with signs of mechanical damage, incorrect maturity, physical damage, or disease were discarded, and the remaining fruits were standardized according to size, shape, and visual uniformity of color. They were then surface disinfected for 1 min with sodium hypochlorite solution (200 mg L^–1^), and rinsed with distilled water (Lima Oliveira et al., 2018).

### Preparation of the Emulsion

A commercial CS-hydrochloride-based formulation (Chitosano; Agrilaete, Italy) was prepared according to the instructions on the product label; the powder product was added to distilled water and dissolved by stirring overnight on a magnetic stirrer. The REO was obtained from Kräuter SAS (Bogotá, Colombia).

To prepare the CS and/or REO emulsions, the methodology reported by Peralta-Ruiz et al. (2020a) was followed, with some modifications. Here, 0.75 ml glycerol per g CS was initially added to 0.5% (w/v) CS as a plasticizer, followed by thorough agitation of the solution. Triton X-100 (Sigma-Aldrich, Germany) was used as an emulsifier, by incorporation at 1% (v/v) vs. REO. Finally, the REO was added directly into the CS hydrochloride dispersion under agitation, to obtain an emulsion with a final concentration of 0.5% (v/v) REO. The REO alone emulsion was prepared following the same procedure above but without CS.

### Postharvest Treatments

Papaya fruits without apparently visual damage were randomly divided into four groups and treated with the emulsions using a concentration of REO of 0.5%. This concentration was used because in previous work we observed that this amount had a sublethal effect on *Colletotrichum gloeosporioides* (Peralta-Ruiz et al., 2020a). Three replicates of 20 fruits were used for each treatment and the control. The papayas were carefully coated with the emulsion following the relevant treatment (0.5% CS, 0.5% REO, and 0.5% CS-REO) by their complete immersion for 2 min, to the control samples was used water. The concentrations of CS were selected because, in a preliminary experiment, we observed a significant reduction of *C. gloeosporioides*. The fruits were then air-dried and kept in plastic boxes at 25 ± 1°C and 62% relative humidity for 9 days.

### Decay Evaluation

To determine the numbers of decayed fruit after the storage period (9 days), we used the relative decay measurement (number of decayed fruit/number of total fruit) for each treatment. Decay severity (*DS*) was also calculated according to the methodology proposed by Romanazzi et al. (2013), following an empirical 0–5 rating scale according to the fruit surface infected: 0, healthy fruit; 1, 1–20% infected; 2, 21–40% infected; 3, 41–60% infected; 4, 61–80% infected; 5, ≥81% infected. McKinney’s disease index (*MI*) was calculated according to Eq. (1) (McKinney, 1923):


(1) $$MI=\left[\frac{1n+2n+3n+4n+5n}{N\times D}\right]\times 100$$

Where *n* is the number of fruit classified in each category of DS, N is the total number of fruit examined (i.e., healthy and infected), and D is the highest category of DS that occurred on the empirical scale used.

The *in situ* effects of the CS-REO combination were determined using Abbott’s equation for synergy calculation, following the method reported by Rahman et al. (2014), with some modifications. First, the protection index (*PI*) was calculated for the DS for each treatment, according to Eq. (2):


(2) $$\mathrm{PI}=\frac{\left({\text{DS}}_{\mathrm{control}}-{\text{DS}}_{\mathrm{treatment}}\right)}{{\text{DS}}_{\mathrm{control}}}\times 100$$

Then, the expected efficacy (*E*_*exp*_) was calculated according to Eq. (3):


(3) $${\text{E}}_{\exp}={\text{PI}}_{\mathrm{CS}}+{\text{PI}}_{\mathrm{REO}}-\left(\frac{{\text{PI}}_{\mathrm{CS}}\times {\text{P}}_{\mathrm{REO}}}{100}\right)$$

The synergistic effects (Abbott index; *AI*) were calculated according to Eq. (4):


(4) $$\mathrm{AI}=\frac{{\text{E}}_{\mathrm{obs}}}{{\text{E}}_{\exp}}$$

Where E_obs_ is the PI determined for the 0.5% CS-REO treatment. A synergistic effect was assigned for *AI* ≥ 1.5, an additive effect for 0.5 ≤ *AI* < 1.5, and an antagonistic effect for *AI* < 0.5 (Peralta-Ruiz et al., 2020a).

### Gene Expression Analysis

To assess the ability of treatments to induce defense response on the papaya fruit, the relative gene expression by reverse transcription quantitative real-time PCR (RT-qPCR) method was performed according to Minimum Information for Publication of Quantitative Real-Time PCR Experiments (MIQE) guidelines (Bustin et al., 2009).

#### Sample Treatment

The gene expression study for the papaya fruit was performed according to the four different treatments (control-water, 0.5% CS, 0.5% REO, and 0.5% CS-REO), previously described. After the treatments, the fruits were arranged in plastic boxes and stored for 0.5, 6, 24, 48, and 72 h, at 25°C and 95–98% relative humidity. At each time, three fruits per treatment were peeled to a thickness of ∼5 mm using a potato peeler, thus, removing the epicarp (outer skin) and some of the mesocarp (edible part). The fruit tissue samples for each treatment (30 g) were frozen in liquid nitrogen and stored in plastic bags at −80°C until RNA extraction. The experiments were repeated at least twice.

#### RNA Extraction

High-quality total RNA was obtained from the fruit following the methodology of Landi et al. (2014). Briefly, 30 g papaya fruit tissue was ground in liquid nitrogen, and 400 mg of the resulting fruit powder was randomly collected for RNA extraction. Extraction buffer was added [1 ml; 100 mM Tris–HCl, pH 8.0, 25 mM ethylenediaminetetraacetic acid disodium salt (EDTA), pH 8.0, 2% (w/v) hexadecyltrimethylammonium bromide (CTAB) (Sigma), 2% (v/v) β-mercaptoethanol, 2.5 M NaCl, 2% (w/v) soluble polyvinylpyrrolidone-40 (PVP-40)], and the samples were incubated at 65°C for 40 min. The supernatants were then transferred to new tubes with an equal volume of chloroform/isoamyl alcohol (24:1) and mixed and centrifuged at 10,000 × *g* for 8 min at 4°C. This last step was repeated two more times. The total RNA was precipitated in 0.25 vol. 10 M LiCl, and kept overnight at 4°C. The samples were then centrifuged at 10,000 × *g* for 30 min at 4°C, washed in 70% ethanol, dried, and resuspended in 50 μl double-distilled diethyl pyrocarbonate water. The RNA quality was determined based on an absorbance ratio of 1.80 to 2.00 at 260/280 nm, and 1.5 to 2.0 at 260/230 nm, using a spectrometer (BioPhotometer plus; Eppendorf Inc., Westbury, NY, United States).

#### Reverse Transcription

First-strand cDNA was synthesized using iScript TM cDNA synthesis kits (Bio-Rad Laboratories, Hercules, CA, United States) from 40 ng RNA, according to the instructions of the manufacturer. From the RNA of each biological replicate, the cDNA synthesis was performed twice, with the products (20 μl each) mixed and diluted (1/10) according to preliminary tests, with an aim to have an adequate quantity of cDNA to analyze all the selected genes.

#### Primer and Reference Gene Selection

Primers were designed using Primer3 software 7^1^, according to 17 key target genes that code for enzymes linked to signaling pathways that regulate plant defense, pathogenesis-related (PR) protein, cell wall-degrading enzymes, control of redox, abiotic stress, and secondary metabolism of the phenylpropanoid pathway. To screen the most stable reference genes, four housekeeping genes, *18S ribosomal RNA* (*18S-RNA*), α*-tubulin* (*tub*), *elongation factor 1* (*tuf*), and *histone H1* (*H1*), were selected. The genes were identified from the specific sequence of *C. papaya* deposited in National Center for Biotechnology Information (NCBI) GenBank. The main functions of the genes and the related coding enzymes analyzed in this study are reported in Table 1. The primer pairs were chosen and validated *in silico* using primer BLAST specific analysis^2^, and then according to the melting profiles obtained by RT-qPCR, as described later. The stabilities of candidate reference genes were evaluated using algorithms: geNorm module of qbase + (Biogazelle) (Vandesompele et al., 2002). These algorithms rank the reference genes based on the stability value (*M*-value). A lower *M*-value corresponds to a more stable gene. The recommended stability for homogenous samples is *M*-value < 0.5 [coefficient of variation, (CV) < 0.25]; and for heterogeneous samples is *M*-value < 1 (CV < 0.5) (Bustin et al., 2009).

**TABLE 1:** Genes selected for gene expression.

Gene name	Abbreviated	NCBI code	Function
*Salicylic acid binding protein 2*	*SABP2*	XM_022039404.1	Required to convert methyl salicylate to salicylic acid; part of signal transduction pathways that activate systemic acquired resistance in systemic tissue (Park et al., 2009)
*Suppressor of npr1-1, constitutive 1*	*SNC1*	XM_022056966.1	Disease resistance protein involved in salicylic acid dependent defense response pathway. Triggers a defense system that promotes programmed cell death (Zhu et al., 2010)
*Pathogenesis related protein 1*	*PR-1*	XM_022048043.1	Involved in defense reactions of plants against pathogens. Long been used as marker for salicylic-acid-mediated disease resistance (Gao et al., 2015)
*Jasmonate O-methyltransferase*	*JMT*	XM_022037106.1	Catalyzes methylation of jasmonate into methyl jasmonate. Acts as cellular regulator in different processes and defense responses (Seo et al., 2001)
*Linoleate 13S-lipoxygenase 2-1, chloroplastic*	*LOX2*	XM_022052808.1	Involved in diverse aspects of plant physiology, including pest resistance and senescence. Involved in bulk production of jasmonate upon wounding (Bannenberg et al., 2009)
*Ethylene receptor, transcript variant X2*	*ETR2*	XM_022038539.1	Related to bacterial two-component regulators. Acts as negative regulator of ethylene signaling (O’Malley et al., 2005)
*Ethylene responsive transcription factor RAP2-13*	*RAP2-13*	XM_022041977.1	Probably acts as transcriptional activator. Binds to pathogenesis related promoter element. Maybe involved in regulation of gene expression by stress factors (Paul et al., 2016)
*Peroxidase 10*	*PRX10*	XM_022052459.1	Removal of H_2_O_2_, oxidation of toxic reductants, biosynthesis and degradation of lignin, auxin catabolism, response to oxidative stresses, wounding, and pathogen attack (Rhee et al., 2001)
*Pathogenesis related protein 5*	*PR-5*	XM_022040713.1	Involved in response to pathogens (El-Kereamy et al., 2011)
*Chitinase 2*	*Cht2*	XM_022055626.1	Encodes chitinase-like protein expressed predominantly in stems (Hossain et al., 2010)
Endo-1,3;1,4-beta-D-glucanase	*GLUC*	XM_022049329.1	Role in control of plant growth. Mediates specific degradation of cell wall (Thomas et al., 2000)
*Polygalacturonase*	*PG*	XM_022056889.1	Important pectolytic glucanase, primarily implicated in softening of fruit during ripening (García et al., 2009)
*NAC domain protein*	*NAC*	XM_022052621.1	Transcription factors highly responsive to abiotic stresses. NACs have roles in maintaining water status under drought or salt conditions (Lv et al., 2016)
*Heat shock cognate 70 kDa protein 2*	*HSP70*	XM_022054737.1	In cooperation with other chaperones, key components that facilitate folding of *de novo* synthesized proteins; also responsible for degradation of damaged proteins under stress (Ahn et al., 2005)
*Anthocyanidin 3-O-glucosyltransferase*	*UFGT*	XM_022051791.1	Participates in flavonoid biosynthesis; involved on defense against pathogen attack (Hu et al., 2011)
*Flavonol synthase*	*FLS*	XM_022056718.1	Participates in flavonoid biosynthesis; involved in defense against pathogen attack (Hammerbacher et al., 2019)
*Phenylalanine ammonia-lyase*	*PAL*	XM_022032339.1	Key enzyme in phenol synthesis pathway; considered primary inducible response in plants against several biotic and abiotic stresses (Kim and Hwang, 2014)
***Elongation factor 1***	***Tuf***	XM_022042067.1	Responsible for enzymatic delivery of aminoacyl tRNAs to ribosomes, (Sasikumar et al., 2012)
***α-tubulin***	***Tub***	XM_022035406.1	Polymerizes into long chains or filaments that form microtubules; hollow fibers that serve as skeletal system for living cells (Janke and Magiera, 2020)
***Histone H1***	***H1***	XM_022052470.1	Dominant role in establishing compaction state of nucleosomes and influencing conformation (Woodcock et al., 2006)
***18S ribosomal RNA***	***18S-RNA***	U42514.1	Active center of protein synthesis in 40S ribosomal subunit (Poltronieri and Hong, 2015)

*The main functions of the gene products are also given. Bold, reference genes.*

#### Quantitative Real-Time PCR

The RT-qPCR reactions were carried out in triplicate in a total volume of 12 μl each, which contained 5.6 μl diluted cDNA, 0.20 μM of each primer, and 6 μl SsoAdvanced Universal SYBR Green Supermix, using a real-time detection system (CFX Connect; Bio-Rad Laboratories). The cycling conditions were as follows: 4 min denaturation at 95°C, followed by 40 cycles at 95°C for 20 s, and 60°C for 40 s. Melting curve analysis was performed over the range of 65–98°C. All of the assays included no-RT and no-template controls to determine the nonspecific amplification. The RT-qPCR efficiency (*E*) of each primer pair was determined using standard curves generated according to *E* = 10 – 1/slope. The diluted cDNAs from samples (10 μl each) were mixed, and then four serial dilutions 1:5, (initial dilution, 0.2, 0.04, 0.008) were obtained. For each primer pair, the standard curve was generated from two technical replicates.

#### Statistical Analysis

For disease incidence, each experiment was repeated at least twice, using a completely randomized block design. The normality of the data was tested using Shapiro–Wilk tests, and the homogeneity of the variances was tested using Levene’s test, using STATISTICA ver. 13.0 (TIBCO Inc., Palo Alto, CA, United States). Appropriate transformations were determined using the Skewness coefficient. The arcsine of the square root of the proportion was applied to the disease incidence data.

Relative changes in gene expression data were determined using the 2^–ΔΔCt^ method (Livak and Schmittgen, 2001), normalized using the reference genes selected in this study, and compared to the untreated control at 0.5 hpt. Each gene was analyzed with three technical replicates for each of the two biological replicates (*n* = 6). To evaluate both the effects of the coatings on the papaya fruit and the gene expression variations in response to the treatments, the data from each sampling point were shown as means ± SD and were statistically evaluated using ANOVA, followed by individual comparisons using Duncan’s multiple range tests, with significance set at *p* ≤ 0.05. For each treatment at each time point, the relative fold-changes were calculated to relevant controls and shown in the heatmap^3^.

## Results

### Fruit Decay

**FIGURE 1:**
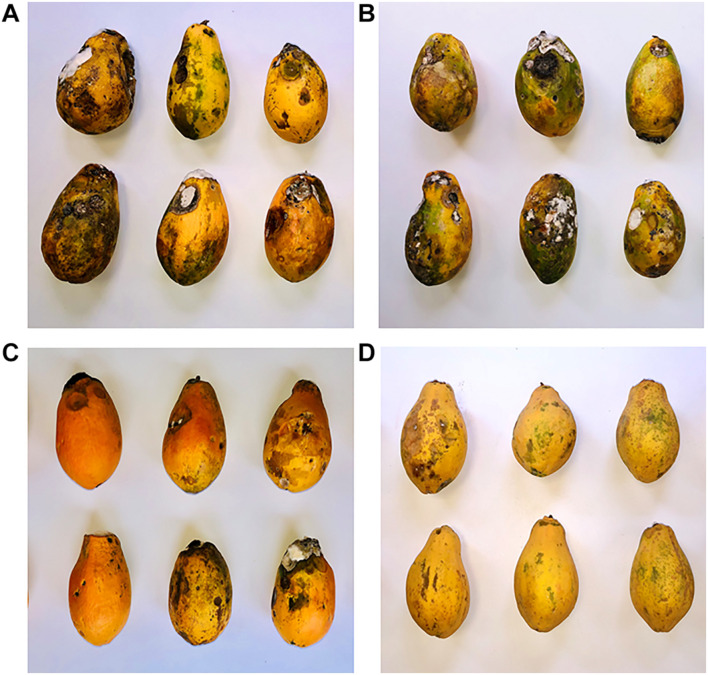
Representative images of incidence of postharvest decay in papaya fruit following 9 days of storage at 25°C ± 1°C after the treatments with water (**A**; control), 0.5% chitosan (CS) **(B)**, 0.5% *Ruta graveolens* essential oil (REO) **(C)**, and their combination 0.5% chitosan, 0.5% *Ruta graveolens* essential oil (CS-REO) **(D)**.

The effects of the treatments with emulsions of 0.5% CS, 0.5% REO, and their combination, CS-REO, on the incidence and severity of the papaya fruit decay over 9 days of storage at 25°C are reported in Table 2 and illustrated in Figure 1. The incidence of decay with 0.5% CS was not statistically different from that of the control fruit. In contrast, for both REO and CS-REO treatments, the decay incidence compared to the control was reduced by 21 and 37%, respectively. The severity of the postharvest decay was reduced compared to the control for all of the treatments; for CS by 22%, for REO by 29%, and CS-REO by 44%. The greatest reduction in the McKinney’s index was seen for the combined treatment (CS-REO; 50%). The AI for this combined treatment showed an additive effect between these 0.5% CS and 0.5% REO emulsions when applied together to the papaya fruits.

**TABLE 2:** Effects of the chitosan (CS), *Ruta graveolens* essential oil (REO), and CS-REO treatments on the incidence and severity of the papaya fruit decay after 9 days of storage at 25 ± 1°C.

Treatment	Disease incidence (%)	Disease severity (1–5)	McKinney’s index (%)	Protection Index (%)	Abbott Index
Control (water)	95.0 ± 10.0 a	4.5 ± 0.38 a	90 ± 7.7 a	–	–
0.5 % CS	84.5 ± 11.9 ab	3.5 ± 0.11 b	70 ± 2.3 b	21.8 ± 5.6 b	–
0.5 % REO	75.4 ± 11.1 b	3.2 ± 0.25 b	63 ± 5.0 b	29.4 ± 8.6 b	–
0.5 % CS+REO	60.0 ± 0.0 c	2.5 ± 0.11 c	50 ± 2.3 c	44.3 ± 2.5 a	1.0

*Data are means ± SD.*

*Different letters within columns indicate significant differences between treatments (p ≤ 0.05; Duncan’s multiple range tests).*

### Gene Expression Analysis

For this study, RT-qPCR was set up to analyze the papaya fruits treated with 0.5% CS, 0.5% REO, separately and combined. The melt peak analysis demonstrated a single homogenous peak for all of the primer sets (data not shown), which confirmed the specificity of the amplicons produced in the RT-qPCR for each of the 21 target and reference genes examined (Table 3). No amplification was seen in any of the control (water treatment) assays, which confirmed that the samples were free of contamination with genomic DNA or RNA, or the cDNA template (data not shown). Standard curves using a mix of cDNA samples from the papaya fruit were constructed using four points of five-fold serial dilutions of cDNA, which yielded efficiencies that ranged from 90 to 110% (Bustin et al., 2009; Table 3).

**TABLE 3:** Primer pairs were selected for gene expression analysis and related PCR amplification efficiencies data.

Gene code	Primers (5′forward/5′reverse)	Amplicon size (bp)	Melt curve peak (°C)	PCR efficiency (%)	*R*^2^ for standard curve
*SABP2*	5′gataggcccggttggtattt/5′aagggcatcatgagatttgg	172	80.0	101.8	0.997
*SNC1*	5′ctgattccgtgcttgttgaa/5′taccccaaattcccaccata	171	79.5	106.7	0.983
*PR-1*	5′tctcacttgggacaccactg/5′atgcccacaaacttttccag	220	88.5	99.6	0.997
*JMT*	5′attgcagacctgggttgttc/5′ggaacctgcaattccagaaa	234	82.5	107.2	0.998
*LOX2*	5′ctccgtgcatgctgtttcta/5′tcaacgctaacaagctccaa	207	84.5	98.9	0.998
*ETR2*	5′cgcttgaaagaggaagcact/5′aaactgcacaaggacccatc	211	82.0	97.1	0.984
*RAP2-13*	5′ccaagaaccgtacccgtcta/5′cagacctttgcttcccagag	249	87.0	107.1	1.000
*PRX10*	5′cagcaaacaaagatggagca/5′gatcgggacacgttttctgt	249	85.0	102.1	0.998
*PR-5*	5′ctcagagcacggagaaggac/5′tactcggccgtgttaaaagc	214	89.0	107.7	1.000
*Cht2*	5′gatcctgacagtggcaatca/5′ccacaggcgtgttacgttta	227	81.0	107.8	0.991
*GLUC*	5′tctctcatttccctcgcatt/5′cgacgacgtaggtgtcaaga	261	83.5	108.7	0.998
*PG*	5′tggtggtgcgtatagatgga/5′tgttccgggagttgagaaac	213	85.5	98.54	1.000
*NAC*	5′ggatcgggtatgaagagcaa/5′atttggggctcttcctttgt	280	84.5	106.1	0.997
*HSP70*	5′gagaagtgcttgagggatgc/5′gtacagcagcaccataggc	176	84.0	102.8	0.997
*UFGT*	5′gatgaatcgcagctgaaaca/5′agatcgaattccacccacag	244	88.0	97.6	0.995
*FLS*	5′tgatagccgatgagctgttg/5′acaccgagaaccaaatcagg	189	81.5	103.6	0.998
*PAL*	5′tgttgcagggctattcagga/5′ccaccatcgattccagcaag	238	82.0	101.3	0.997
***tuf***	5′ctggaaagtcgaccaccact/5′aggggcatcaataacagtgc	227	84.0	102.0	0.996
***tub***	5′gagcacactgatgtggcagt/5′ggaacaaggttggtctggaa	197	80.5	104.5	0.987
***H1***	5′acatggaagggaagcacaag/5′cgacttcttcgaggtcttgg	179	83.5	102.7	0.986
***18S-RNA***	5′agaaacggctaccacatcca/5′acccaaggtccaactacgag	247	83.5	102.8	0.997

*PCR amplicon size, amplification efficiencies, and regression coefficients for the standard curves are reported for each primer pair. Bold, reference genes.*

The four putative candidate reference genes *18S-RNA*, *tub*, *tuf*, and *H1* were validated according to the geNorm method, and they showed different stability values. The lowest *M*-values, which correspond to the most stable genes, across all of the treatments tested in this study were seen for the *tub* (0.3357 ± 0.046; CV, 0.1352 ± 0.098) and *H1* (0.3775 ± 0.073; CV, 0.1881 ± 0.078). This indicated that these two reference genes were suitable for the RT-qPCR investigation into these treated papaya fruits. In contrast, *18S-RNA* and *tuf* showed greater instabilities according to the *M*-values (0.7264 ± 0.124; CV, 0.4012 ± 0.072; 0.6354 ± 0.12; CV, 0.5147 ± 0.101; respectively).

The gene expression data were discussed according to their relative fold-changes compared to relevant controls.

#### Genes Involved in Signaling Pathways That Regulate Plant Defense

**FIGURE 2:**
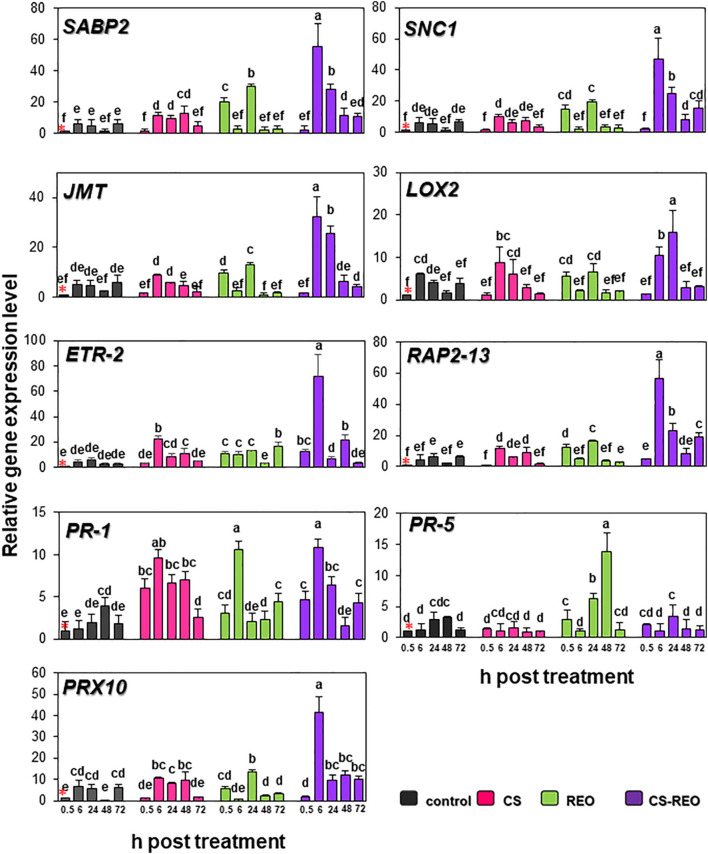
Quantification of relative gene expression of nine of the defense genes examined (as indicated; see also Table 1) in papaya fruit following treatments with water (control), 0.5% CS, 0.5% REO, and their combination (CS-REO). Each experimental replicate represents three technical replicates (*n* = 6). Gene expression given was relative to control at 0.5 h posttreatment, also indicated with a red asterisk, according to the 2^–ΔΔCt^ method (Livak and Schmittgen, 2001). Data are the means ± SD. Columns with different letters are significantly different (*P* ≤ 0.05; Duncan’s multiple range tests).

**FIGURE 3:**
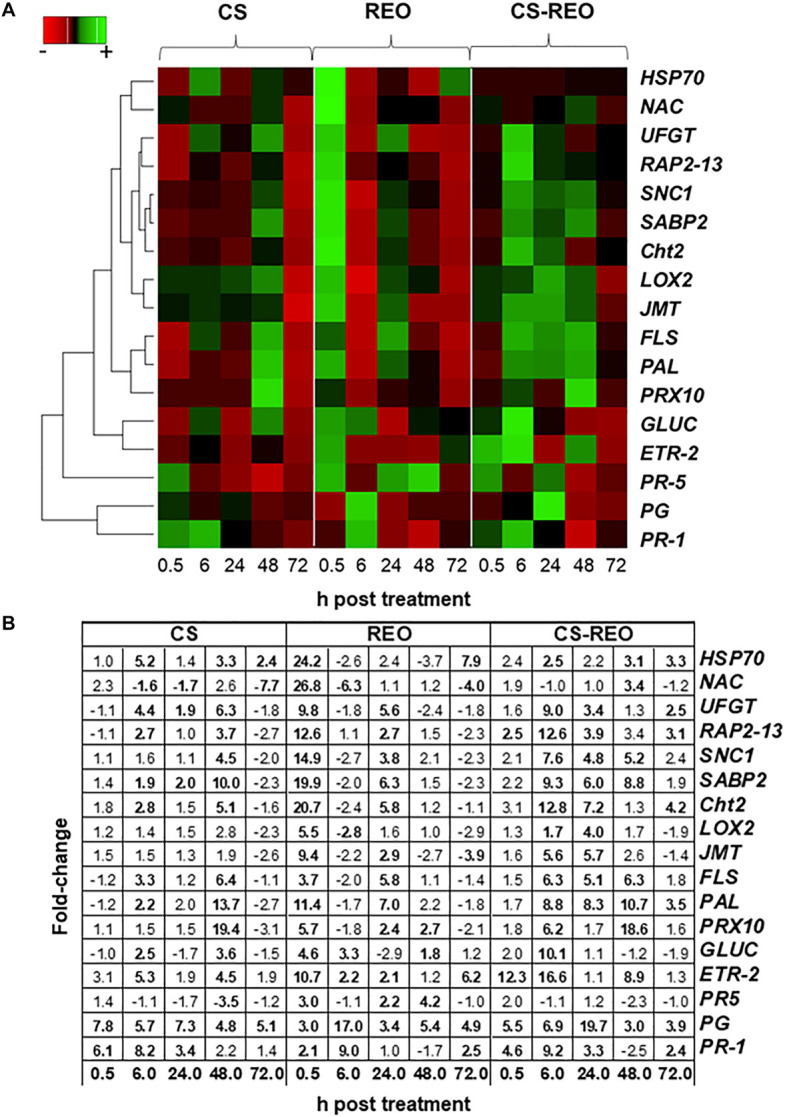
Gene expression heatmap. Hierarchical clustering according to Pearson’s correlation similarities and average linkage of the defense genes examined (as indicated; refer to Table 1) in papaya fruit following treatments with 0.5% CS, 0.5% REO, and their combination (CS-REO). For each gene, the maximum (green color) and minimum (red color) fold-changes are compared to the control (water) treatment at 0.5, 6, 24, 48, and 72 h posttreatment **(A)**. The average fold-change values compared to the control (water) treatment at 0.5, 6, 24, 48, and 72 h posttreatment, used for hierarchical clustering, were shown. In bold, the data significantly different (*P* ≤ 0.05; Duncan’s multiple range tests), compared to relevant controls were indicated **(B)**.

*SABP2* is involved in the salicylic acid (SA) pathway. Its levels of expression in the fruit treated with CS showed moderate upregulation at 6 hpt and 24 hpt, of ∼2-fold, compared to the relevant control. However, its greatest upregulation was at 48 hpt, at 9.9-fold. After REO treatment, *SABP2* expression increased more rapidly, to initially peak at 0.5 hpt at 19.9-fold, and then increased again to 6.3-fold at 24 hpt. Then for CS-REO, *SABP2* showed increased expression at 6, 24, and 48 hpt of 9.2-fold, 5.9-fold, and 8.7-fold, respectively, with respect to the control (Figures 2, 3). Expression of the *SNC1* gene suppressor of NPR1 was correlated with *SABP2* expression. Indeed, after CS treatment, *SNC1* expression increased at 48 hpt to 4.5-fold, while after REO treatment, it was upregulated at 0.5 hpt by 14.9-fold and at 24 hpt by 3.8-fold. Finally, after CS-REO treatment, *SNC1* showed increased expression at 6, 24, and 48 hpt, of approximately 5-fold to 7.5-fold (Figures 2, 3). The *JMT* gene is a part of the jasmonate pathway, and its expression levels in the papaya fruits were not affected by CS treatment, while REO increased *JMT* expression at 0.5 hpt by 9.4-fold, and at 24 hpt by 2.9-fold. *JMT* was upregulated with the CS-REO treatment at 24 and 48 hpt, by 5.6-fold for both (Figures 2, 3). Like for *JMT*, the *LOX2* gene participates in jasmonate synthesis, and its expression was also not affected by CS. Instead, after REO treatment, this transcript was upregulated at 0.5 hpt by 5.5-fold, followed by downregulation at 6 hpt of −2.8-fold. The CS-REO treatment upregulated *JMT* at 6 and 24 hpt, by 1.7-fold and 4-fold, respectively (Figures 2, 3). For the genes involved in ethylene (ET) transcription, the expression of both *ETR-2* and *RAP2-13* was upregulated after CS treatment mainly at 6 and 48 hpt: for *ET-2*, by 5.2-fold and 4.5-fold, respectively, and for RAP2-13, by 2.6-fold and 3.6-fold, respectively (Figures 2, 3). After REO treatment, *ETR-2* and *RAP2-13* showed similar expression profiles, with strong upregulation at 0.5 hpt, of 10.7-fold and 12.6-fold, respectively (Figures 2, 3). Then, *ETR-2* was upregulated at 6 and 24 hpt by 2-fold and at 72 hpt by 6-fold, while at 24 hpt, *RAP2-13* was upregulated by 2.7-fold (Figures 2, 3). For CS-REO treatment, there was upregulation of *ETR-2* at 0.5, 6, and 48 hpt of 12-fold, 16-fold, and 8.8-fold, respectively, while *RAP2-13* expression was upregulated at 0.5 hpt by 2.4-fold, at 6 hpt by 12.6-fold, and at 24 and 72 hpt by about 3-fold (Figures 2, 3).

#### Genes Involved in Oxidative Stress

The CS treatment upregulated *PRX10* expression at 48 hpt by 19.4-fold, while REO treatment showed upregulation at 0.5, 24, and 48 hpt by 5.6-fold, 2.4-fold, and 2.6-fold. The CS-REO combination promoted a moderate increase in *PRX10* expression at 0.5 hpt, of 1.8-fold, while greater upregulation was seen at 6 hpt and 48 hpt, of 6.8-fold and 18.6-fold, respectively (Figures 2, 3).

#### Genes for PR Proteins

For the *PR-1* gene, its expression increased after both CS and CS-REO treatments, mainly at 0.5, 6, and 24 hpt, at 6.1-fold, 8.1-fold, 3.4-fold, and 4.6-fold, 9.1-fold, and 3.2-fold, respectively. The *PR-1* gene up-regulation was observed also at 72 hpt by 2.4-fold by CS-REO. After the REO treatments, *PR-1* expression increased mainly at 6 hpt, by 9-fold, and at 72 hpt, by 2.4-fold (Figures 2, 3). The *PR-5* gene was upregulated only after REO treatment, and at 0.5, 24, and 48 hpt, by 2.9-fold, 2.1-fold, and 4.1-fold. The downregulation was observed at 48 hpt by 3.5-fold by CS, were no changes in *PR-5* gene expression with CS-REO treatments (Figures 2, 3).

#### Genes for Cell Wall-Degrading Enzymes

**FIGURE 4:**
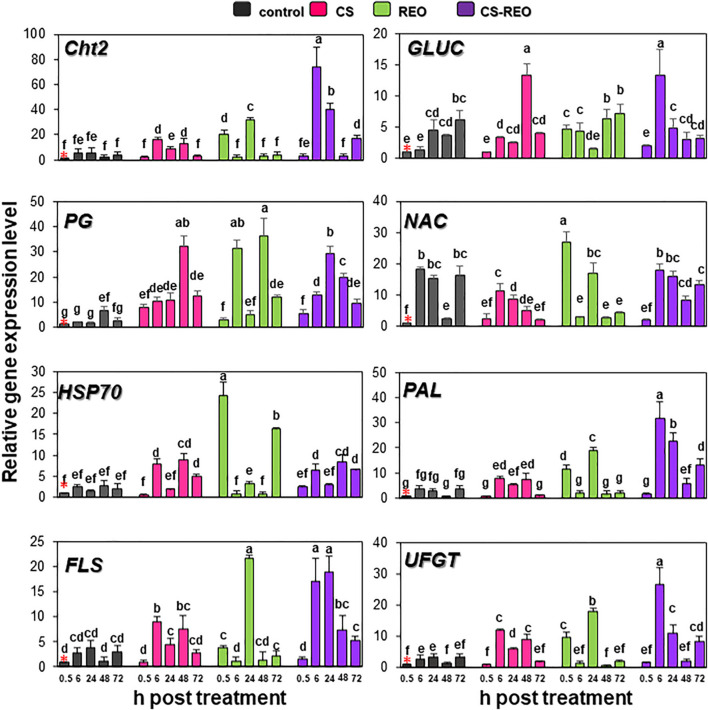
Quantification of relative gene expression of the remaining eight of the defense genes examined (as indicated; refer to Table 1) in papaya fruit following treatments with water (control), 0.5% CS, 0.5% REO, and their combination (CS-REO). Each experimental replicate represents three technical replicates (*n* = 6). Gene expression given was relative to control at 0.5 h posttreatment, also indicated with a red asterisk, according to the 2^–ΔΔCt^ method (Livak and Schmittgen, 2001). Data are the means ± SD. Columns with different letters are significantly different (*P* ≤ 0.05; Duncan’s multiple range tests).

The expression of *Cht2* was upregulated after CS treatment at 6 and 48 hpt, by 2.8-fold and 5-fold, respectively; conversely, after REO treatment, the gene was upregulated at 0.5 and 6 hpt, by 20.7-fold and 5.8-fold, respectively. The CS-REO treatment combination increased *Cht2* expression at 6, 24, and 72 hpt, by 12.8-fold, 7.21-fold, and 2.5-fold (Figures 3, 4).

Similar to *Cht2*, after CS treatment, *GLUC* expression increased at 6 and 48 hpt, 2.4-fold, and 3.5-fold, respectively. The REO treatment upregulated *GLUC* at 0.5, 6, and 48 hpt by 4.6-fold, 3.3-fold, and 1.8-fold, respectively, while CS-REO upregulated *GLUC* mainly at 6 hpt, by 10-fold (Figures 3, 4).

The *PG* gene was upregulated after all of the treatments at all of the time points by >3.8-fold, except for REO at 0.5 hpt. However, the greatest *PG* expression after CS was at 0.5 hpt, at 7.7-fold, while for both REO and CS-REO treatments, high *PR* expression was also seen at 6 hpt, at 16.9-fold, and 19.5-fold, respectively (Figures 3, 4).

#### Genes Involved in Abiotic Stress

After CS treatment, the *NAC* gene was downregulated at 6, 24, and 72 hpt, at −1.6-fold, −1.7-fold, and −7.7-fold, respectively (Figures 3, 4). Instead, REO treatment strongly increased *NAC* expression at 0.5 hpt at 26.8-fold, followed by downregulation at 6 and 72 hpt at −6.25-fold and −4-fold (Figures 3, 4). In contrast, CS-REO affected *NAC* expression only at 48 hpt, when it was upregulated by 3.4-fold (Figures 3, 4).

The *HSP70* expression after CS treatment was upregulated at 6, 48, and 72 hpt by 5.2-fold, 3.3-fold, and 2.4-fold, respectively, while, after REO treatment, this occurred at 0.5 and 72 hpt, by 24.2-fold and 7.9-fold, respectively. With CS-REO treatment, *HSP70* was upregulated at 6, 48, and 72 hpt by 2.5-fold to 3.3-fold (Figures 3, 4).

#### Genes Involved in the Phenylpropanoid Pathway

The *PAL*, *FLS*, and *UFGT* genes are all linked to the phenylpropanoid pathway, and they showed similar gene expression patterns.

In more detail, CS treatment increased *PAL* expression at 6 and 48 hpt by 2.1-fold and 13.7-fold, while REO treatment upregulated *PAL* at 0.5 and 24 hpt by 11.4-fold and 6.9-fold. Then after treatment with the CS-REO combination, this increased *PAL* at 6, 24, 48, and 72 hpt by 8.7-fold, 8.3-fold, 10.7-fold, and 3.5-fold, respectively.

The *FLS* gene was upregulated by CS at 6 and 48 hpt, at 3.3-fold and 6.4-fold, by REO at 0.5 and 24 hpt, at 3.7-fold and 5.8-fold, and by CS-REO at 6, 24, and 48 hpt, at 6.5-fold, 5.0-fold, and 6.2-fold.

Finally, the *UFGT* gene was upregulated by CS at 6, 24, and 48 hpt by 4.4-fold, 1.9-fold, and 6.3-fold, and by REO at 0.5 and 24 hpt by 9.8-fold and 5.6-fold. The CS-REO treatment upregulated *UFGT* at 6, 24, and 72 hpt by 9.0-fold, 3.4-fold, and 2.4-fold (Figures 3, 4).

## Discussion

In this study, we evaluated the effectiveness against postharvest anthracnose in papaya fruit of commercial formulations of CS and REO, both individually and combined. Furthermore, we also investigated the effects of these treatments on the activation of the transcription of key genes involved in plant defense mechanisms. In these fruits under postharvest storage at room temperature, and compared to the CS and REO treatments, the CS-REO combination indeed showed the greatest control of fungal decay and severity, as also for the McKinney’s Index. Similar results were observed in previous studies on papaya artificially inoculated with *C. gloeosporioides*, where the fruit treated with CS- 0.5% REO showed an incidence decay of 60% and a lower severity lesion (Peralta-Ruiz et al., 2020a). According to the AI, CS-REO showed an additive effect from CS and REO alone, as also indicated by Peralta-Ruiz et al. (2020a). Our findings agree with several previous studies that have reported on synergistic or additive effects of CS and EOs in combination for fruit preservation (Yuan et al., 2016; Lima Oliveira et al., 2018; de Oliveira et al., 2020; Elshamy et al., 2021).

The antifungal properties of CS are usually related to the interactions of its positive amino groups with the fungal membrane, which can induce changes to the permeability of the plasma membrane (Grande-Tovar et al., 2018). Also, the barrier effect of CS inhibits the germination of fungal spores and reduces the fungal decay on fruit (Romanazzi et al., 2018; Rajestary et al., 2021). This can preserve the physicochemical properties of the fruit for longer, thus also prolonging the shelf life (Nair et al., 2020).

In the present study, the effects of CS were reinforced by the addition of REO, which has been observed for other EOs (Sivakumar and Bautista-Baños, 2014; Huang et al., 2021), through their antimicrobial, antifungal, and antioxidant activities (Grande Tovar et al., 2019; González-Locarno et al., 2020; Peralta-Ruiz et al., 2020a; Zhang D. et al., 2020). It has been reported that the mode of action of such EOs is based on the cytotoxic effects including the induction of cell death by activation of apoptosis and/or necrosis processes (Nazzaro et al., 2017; Sharifi-Rad et al., 2017). Indeed, recent studies have reported that the fungal membrane of *C. gloeosporioides* was compromised after 1 h with 1% REO (Peralta-Ruiz et al., 2020a) and that under similar treatment, *Candida* yeast showed irreversible cell membrane damage with increased intracellular leakage of macromolecules (Donadu et al., 2021). In addition to the antimicrobial actions, several studies have shown activities as resistance inducers and/or activators of plant defense mechanisms for both CS (Ali et al., 2012; Landi et al., 2014, 2017; Coqueiro et al., 2015; Obianom et al., 2019) and EOs (Banani et al., 2018; Hou et al., 2020).

In the present study, we investigated for the first time, the expression of a range of plant defense genes induced by CS and REO and their combination in the papaya fruit. We selected genes that are involved in key metabolic pathways of plant defense responses to provide basic information on the main mechanisms involved in these actions of such CS–EOs combinations.

We first validated a useful protocol for this gene expression study in papaya fruit using RT-qPCR. This technique has been widely used to evaluate gene expression because of its speed and sensitivity. However, reliable quantification of gene expression mainly depends on accurate normalization. For this reason, the selection and validation of the reference genes were among the most crucial steps in the setting up of this RT-qPCR (Vandesompele et al., 2002; Bustin et al., 2009; Köhsler et al., 2020). This indicated two new suitable genes for gene expression studies in papaya fruit: *H1* and *tub*. In this papaya fruit investigation, and independent of the treatments used in this study, these genes showed greater stability than *18S rRNA*, as suggested by Zhu et al. (2012), and the *tuf* gene.

Based on this transcript analysis, this study showed that both the 0.5% CS and 0.5% REO treatments affected gene expression in the papaya fruits. However, according to the transcription of the individual genes analyzed in this study, some differences were seen. The jasmonic acid (JA)-responsive genes of *JMT* and *LOX2* (Nakamura et al., 2011) were not affected in these papaya fruits by CS treatment, while they were involved in REO and CS-REO treatments. On the other hand, both CS and REO modulated the expression of genes linked to the SA pathway (i.e., *SABP2*, *PR-1*, and *SNC1*) and ET pathway (i.e., *ET-2* and *RAP2-13*). Also, the *PR-5* gene, which is linked to the SA pathway (Fu et al., 2012; Ali et al., 2018), was upregulated by REO treatment.

Many studies have indicated that the SA-mediated defense signaling pathway is important for activation of pathogen-associated molecular patterns that trigger immunity and for effector-triggered immunity, as well as for systemic acquired resistance (Jones and Dangl, 2006; Walters and Fountaine, 2009; Spoel and Dong, 2012). As well as the SA-mediated defense signaling pathways are indicated as linked to biotrophic pathogen infection, the jasmonate/ET pathways are indicated as involved with regulators of stress responses against necrotrophic fungi (Wasternack and Hause, 2013), and induction of volatile compounds in response to insect herbivores (Rodriguez-Saona et al., 2013) and during abiotic stress (Wang et al., 2020). However, investigations carried out in recent years have shown the complexity of the plant regulatory network against stress. Indeed, cross talk between the SA-dependent, JA-dependent, and ET-dependent signaling pathways is believed to be involved in the fine-tuning of the defense reaction, to lead to the activation of an optimal mix of defense responses to resist any particular pathogen (Pieterse et al., 2009; Jia et al., 2018). In support of this, a previous study showed that oregano EO can trigger the plant’s innate immune system that involves SA, JA, and ET synthesis and signaling, with the activation of PR proteins and phytoalexin synthesis (Rienth et al., 2019).

However, overall, our study showed that both CS and REO, and their combination CS-REO, can trigger signaling defense mechanisms to induce genes involved in phenylpropanoid biosynthesis and in cell wall metabolism, which demonstrates key roles for both secondary metabolite and cell wall genes in postharvest defense pathways (Landi et al., 2014, 2017; Xoca-Orozco et al., 2019; Zhang et al., 2020).

The present study underlines the involvement of genes linked to heat stress tolerance and cellular apoptotic change, in terms of *NAC* and *HSP70* strongly upregulated in the early phase after REO treatment, while mainly for *NAC* gene, downregulation and /or unaffected gene expression was observed according to the other treatments. Part of the core of the data presented here is the difference detected between the CS and REO treatments according to activation times. The CS treatment affected gene expression mainly after 24 and 48 hpt, while the REO treatment strongly upregulated the gene transcripts earlier, at 0.5 hpt, then generally the gene expression drastically decreased at 6 hpt, then increased again mainly at 24 hpt, but to a lesser extent. In both cases, changes in gene expression over time are not surprising, given that gene expression is a complex stochastic process that represents the combination of numerous enzymatic reactions with unknown cell variables (Dal Co et al., 2017; Park et al., 2018). These changes in gene expression have been proposed to occur as the result of an optimization process, due to a trade-off between speed and cost (to the cell) of transcript production (Zaslaver et al., 2004). Also, the changes in gene expression might be linked to adaptation to the changes in stress (Koch and Guillaume, 2020). The present study suggests that these differences in gene expression over time can be correlated to the different natures of these two compounds, CS and REO. EOs are volatile, thermolabile, and unstable, which results in natural fluctuations in their components and compositions. They are highly reactive substances, and their antimicrobial activities might be impaired by changes in pH or temperature (Turek and Stintzing, 2013). On the other hand, CS has excellent film-forming properties, which will provide a mechanical barrier for the control of the respiration rate and decrease the loss of volatiles. The highly reactive volatile EOs can thus be stabilized and incorporated into the biodegradable, nontoxic CS, to produce transparent elastic films that can improve the effectiveness of their postharvest actions. This additive effect was evident in the gene expression patterns. Indeed, the genes analyzed after the CS-REO treatment generally showed greatly increased expression levels starting at 6 hpt, rather than at 0.5 hpt, as observed after the REO treatment. This might be due to the encapsulation of REO within the CS emulsion. Then, later on after the CS-REO treatment (i.e., beyond 6 hpt), for most of the genes, the expression levels were maintained relatively high for longer. This was seen for genes linked to the phenylpropanoid pathway, as *PAL*, *FLS*, and *UFGT*, as well as for genes involved in the signaling pathways that regulate plant defense, as *SABP2*, *SNC1*, *M-JA*, *LOX2*, *ETR-2*, and *RAP2-13*, but not for genes more closely with abiotic stresses such as NAC and HSP70, which suggests greater control of the cell stress by CS. This shows that the increase in the effectiveness of the disease control was associated with a broader and constant physiological change in the levels of the gene transcripts with roles in the induction of postharvest defense responses.

## Conclusion

This study initially confirms that the incorporation of REO into the edible CS coating improves the control of postharvest decay of papaya fruit compared to the use of these treatments individually. For the first time, the main molecular mechanism in the triggering of defense pathways linked to the CS-REO combination is also indicated, as compared to their application. Indeed, CS largely showed effects on genes involved in the regulation of plant defense at 6 hpt, while REO showed strong induction of overexpression in the early phase, at 0.5 hpt. The CS-REO treatment also demonstrated additive actions on gene expression in these papaya fruits. This was supported by the delay in gene upregulation for CS-REO compared with REO, from 0.5 to 6 hpt, and kept longer over time. Indeed, this effect might be associated with CS such that it incorporates the volatile substances of the rue oil, and then releases them more slowly, to improve the regulation of cell stress.

This study thus represents an important first step in our better understanding of the molecular mechanisms involved in the combined effects of CS and EOs for postharvest control of fruit diseases. Similar studies are important for the control of postharvest decay, to suggest new strategies for induction of defense reactions in plants, and their possible use for the production of new active biological preparations.

## Data Availability Statement

The raw data supporting the conclusions of this article will be made available by the authors, without undue reservation.

## Author Contributions

LL and YP-R: methodology, statistical analysis, software, and writing—review and editing. LL, YP-R, CC-L, and GR: conceptualization, design of the study, and review and editing of the manuscript. CC-L and GR: supervision, project administration, and funding acquisition. All authors have read and agreed to the published version of the manuscript.

## Conflict of Interest

The authors declare that the research was conducted in the absence of any commercial or financial relationships that could be construed as a potential conflict of interest.

## Publisher’s Note

All claims expressed in this article are solely those of the authors and do not necessarily represent those of their affiliated organizations, or those of the publisher, the editors and the reviewers. Any product that may be evaluated in this article, or claim that may be made by its manufacturer, is not guaranteed or endorsed by the publisher.

## Footnotes

1
https://bio.tools/primer3

2
http://www.ncbi.nlm.nih.gov/Blast.cgi

3
http://www.heatmapper.ca/expression/
